# ﻿*Chaetocapnodiummagnum* and *Chaetocapnodiumpolonicum* from conifer resins disclose an unknown lifestyle in the Capnodiales (Dothideomycetes)

**DOI:** 10.3897/mycokeys.119.159094

**Published:** 2025-07-14

**Authors:** Paweł Czachura, Paulina Janik, Marcin Piątek

**Affiliations:** 1 W. Szafer Institute of Botany, Polish Academy of Sciences, Lubicz 46, PL-31-512 Kraków, Poland Polish Academy of Sciences Kraków Poland

**Keywords:** Capnodiaceae, conifer resins, morphology, phylogeny, resinicolous fungi, taxonomy, two new species

## Abstract

The paraphyletic order Capnodiales s. lat. was recently divided into eight orders based on phylogeny, morphology, and fungal lifestyle. As a consequence, Capnodiales s. str. was redefined to include fungi called sooty moulds, which grow on sugary exudates (honeydew) excreted by sap-feeding insects. Despite some exceptions, Capnodiales s. str. constitutes a highly specialized order comprising fungi adapted to this specific habitat. The order includes many genera, of which one of the best studied is the genus *Chaetocapnodium*. To date, *Chaetocapnodium* has accommodated 10 species, which are strictly sooty moulds. In this study, two novel species of *Chaetocapnodium* are described from Poland—*Chaetocapnodiummagnum* and *Chaetocapnodiumpolonicum*—based on morphology and multi-gene phylogenetic analyses. Importantly, both species were isolated from resin flows of conifers, which represents a new ecological niche for the genus *Chaetocapnodium* as well as for the entire order Capnodiales s. str. Members of the genus *Chaetocapnodium* were previously reported mostly from the tropics or subtropics (exceptionally from the sub-Antarctic), and therefore they are reported from the temperate zone in Europe for the first time.

## ﻿Introduction

Capnodiales s. lat. was a species-rich order of fungi with taxa representing different lifestyles, inhabiting various substrates, and living in diverse environments (Crous et al. 2009). Recently, molecular phylogenetic analyses showed that the concept of the order was too broad, and consequently it was divided into eight orders: Arthrocatenales, Capnodiales s. str., Cladosporiales, Comminutisporales, Mycosphaerellales, Neophaeothecales, Phaeothecales, and Racodiales (Abdollahzadeh et al. 2020; Piątek et al. 2024). As a consequence, Capnodiales s. str. was restricted to fungi that grow on sugary exudates (honeydew) excreted by sap-feeding insects (Abdollahzadeh et al. 2020). Such fungi are called sooty moulds. Though sooty moulds belong to different phylogenetic lineages within the classes Dothideomycetes and Eurotiomycetes (at the level of species, genera, families, and order) (Hughes 1976; Chomnunti et al. 2014; Flessa et al. 2012, 2021; Piątek et al. 2023, 2024; Czachura and Piątek 2025), the order Capnodiales s. str. is the most speciose and contains almost exclusively this ecological group of fungi (Batista and Ciferri 1963; Hughes 1976; Chomnunti et al. 2014; Abdollahzadeh et al. 2020). However, what is noteworthy is that there are a few species that were reported from different habitats, such as *Kosmimatomycesalatophylus*, described from soil in a salt marsh; *Mycodomusformicartus* (nom. inval.), associated with black ants on bamboo branches; two species of *Readerielliopsis*—*R.fuscoporiae*, described from basidiomata of *Fuscoporiawahlbergii*, and *R.guyanensis*, described from a decaying leaf; and *Tryssglobulusaspergilloides*, described from the underside of leaves of *Banksiamarginata* (Sutton and Pascoe 1987; Decock 2005; Crous et al. 2015, 2020, 2021; Jitjak and Sanoamuang 2019). Moreover, there are reports of fungi isolated from the surface of ant cartons, representing probably undescribed species, residing in Capnodiales s. str. (Voglmayr et al. 2011; Mayer et al. 2023). However, these fungi represent rare exceptions of taxa with different lifestyles within this speciose order, represented almost exclusively by sooty moulds.

Among the strictly sooty mould genera of Capnodiales s. str. is the genus *Chaetocapnodium*. The genus was introduced to accommodate *Chaetocapnodiumsiamense* (Liu et al. 2015). Later, Abdollahzadeh et al. (2020) described five novel species: *Chaetocapnodiumindonesiacum*, *Ch.insulare*, *Ch.summerellii*, *Ch.tanzanicum*, and *Ch.thailandense*, and relocated to this genus two species previously known as *Antennariellaplacitae* (Cheewangkoon et al. 2009) and *Phragmocapniasphilippinensis* (Liu et al. 2015) (now named *Ch.placitae* and *Ch.philippinense*, respectively). Subsequently, Khodaparast et al. (2020) proposed a new combination, *Chaetocapnodiummicroglobulosum*, for *Chaetasbolisiamicroglobulosa* (Batista and Ciferri 1963), while Navarro De La Fuente et al. (2022) described *Chaetocapnodiumzapotae*. In summary, the genus *Chaetocapnodium* presently includes 10 species, and it is one of the best-studied genera within Capnodiales s. str. Members of *Chaetocapnodium* were reported mostly from tropical or subtropical locations in Australia, Indonesia, Iran, Laos, Mexico, the Philippines, Tanzania, and Thailand, and exceptionally from a sub-Antarctic location on Marion Island (South Africa) (Batista and Ciferri 1963; Cheewangkoon et al. 2009; Liu et al. 2015; Abdollahzadeh et al. 2020; Khodaparast et al. 2020; Navarro De La Fuente et al. 2022).

In this study, three fungal strains from resin flows of *Abiesalba* and Larixdeciduassp.polonica in Poland were identified as members of the genus *Chaetocapnodium* based on initial molecular identification using ITS rDNA sequences. After morphological and multi-gene phylogenetic analyses, the strains were identified and described as two novel species of *Chaetocapnodium*.

## ﻿Materials and methods

### ﻿Strains isolation

The strains analyzed in this study were isolated from resin samples of *Abiesalba* and Larixdeciduassp.polonica (Pinaceae), collected in the Modrzyna Reserve in the Beskid Niski Mountains and the Świętokrzyski National Park in Poland. To remove external contaminants, the resin samples were subjected to a washing process as described by Czachura and Janik (2025). Afterwards, resin samples were scraped using razor blades, then resin particles were spread on Petri dishes containing four different media: dichloran–18% glycerol agar (DG18), dichloran rose bengal chloramphenicol agar (DRBC), rose bengal chloramphenicol agar (RBC), and potato dextrose agar (PDA). Mycological media for the isolation of strains from resin samples were prepared according to Crous et al. (2019) and Corry et al. (1995). Initial cultures with resin particles were incubated in the dark at 15 °C or 25 °C. Distinct morphotypes were transferred to malt extract agar (MEA) for preliminary molecular analysis. Among the numerous fungal taxa isolated as described above, three strains representing members of the genus *Chaetocapnodium* were selected for this study. The holotype and additional specimen of *Chaetocapnodiummagnum*, as well as the holotype of *Chaetocapnodiumpolonicum*, are deposited as dried cultures (KRAM F) in the fungal collection of the W. Szafer Institute of Botany, Polish Academy of Sciences, Kraków, Poland, whereas living cultures are deposited in the culture collection (CBS) of the Westerdijk Fungal Biodiversity Institute, Utrecht, the Netherlands, and in the W. Szafer Institute of Botany, Polish Academy of Sciences, Kraków (in the latter case under paraffin oil).

### ﻿Morphological analyses

The macroscopic characterization of the colonies was performed on Petri dishes (Ø 90 mm) with four different media: malt extract agar (MEA), oatmeal agar (OA), potato dextrose agar (PDA), and synthetic nutrient-poor agar (SNA), after two weeks at 15 °C and 25 °C in darkness. Mycological media for morphological analyses were prepared according to Crous et al. (2019). Colors were assessed with support from Rayner’s color chart (Rayner 1970). Micromorphological characterization was examined on SNA using slides or slide cultures under a Nikon Eclipse 80i compound microscope. Photographs were obtained using the NIS‐Elements BR 3.0 imaging software. Figures showing morphological characteristics were prepared using Inkscape 0.92.4.

### ﻿DNA extraction, amplification, and sequencing

DNA was isolated using the CTAB method as described in Czachura and Janik (2025). Amplification was conducted for four loci: the internal transcribed spacer 1 and 2 regions and the intervening 5.8S rRNA gene (ITS), the partial 28S rRNA gene (LSU), the partial RNA polymerase II second largest subunit gene (*rpb2*), and the partial translation elongation factor 1-α gene (*tef1*). Polymerase chain reaction (PCR) mixtures were prepared as described in Czachura and Janik (2025). Primer pairs used for amplification were ITS1 and LR5 for a fragment containing ITS and LSU (White et al. 1990; Vilgalys and Hester 1990), fRPB2-5F and fRPB2-7cR for *rpb2* (Liu et al. 1999), and EF1-983F and EF1-2218R for *tef1* (Rehner and Buckley 2005). PCR amplification conditions for the fragment containing ITS and LSU as well as *rpb2* were set as described in Piątek et al. (2024). Amplification conditions for *tef1* were set as follows: an initial denaturation at 94 °C for 3 min, followed by 40 cycles of amplification (denaturation at 94 °C for 30 s; annealing at 55 °C for 50 s; elongation at 72 °C for 60 s), and a final elongation step at 72 °C for 10 min. The PCR products were purified using Exo-BAP Mix (EURx, Poland). Bidirectional sequencing was carried out by Macrogen Europe B.V. (Amsterdam, The Netherlands). ITS was sequenced with the primer pair ITS1 and ITS4 (White et al. 1990), LSU was sequenced with the primer pair LSU1Fd and LR5 (Vilgalys and Hester 1990; Crous et al. 2009), whereas protein-coding genes were sequenced with the same primer pairs as used for their amplification.

### ﻿Phylogenetic analyses

Sequences were examined, assembled, and trimmed using Geneious Prime® 2022.1.1. Preliminary molecular identification of the analyzed strains was assessed using the megablast search tool in the NCBI GenBank nucleotide database (https://www.ncbi.nlm.nih.gov/genbank/) (Zhang et al. 2000). Phylogenetic analyses were performed using LSU, ITS, *tef1*, and *rpb2* sequences of all known *Chaetocapnodium* species, including selected members of closely related genera (Abdollahzadeh et al. 2020; Khodaparast et al. 2020; Navarro De La Fuente et al. 2022), with *Leptoxyphiumcitri* and *Leptoxyphiummadagascariense* used as the outgroup. Sequences and details of strains used in the phylogenetic analyses are listed in Table 1. Datasets of each molecular marker (LSU, ITS, *tef1*, and *rpb2*) were aligned separately using MAFFT v. 7.490 (Katoh et al. 2002; Katoh and Standley 2013) and concatenated to build a multi-gene matrix using Geneious Prime® 2025.1.2 to construct phylogenetic trees. Phylogenetic reconstruction was performed using maximum likelihood (ML) and Bayesian inference (BI). The best-fit nucleotide substitution models were selected for each partition using ModelTest-NG v. 0.2.0 (Flouri et al. 2014; Darriba et al. 2020), based on the Bayesian information criterion (BIC). The following partitions were specified: LSU, ITS, and the first, second, and third codon positions of protein-coding genes (*tef1* and *rpb2*). The models applied in ML analysis were as follows: HKY+I+G4 for LSU and ITS; GTR+G, F81+I+G4, and F81+I+G4 for the first, second, and third codon positions of *tef1*, respectively; and HKY+I, HKY+G4, and GTR+G4 for the first, second, and third codon positions of *rpb2*, respectively. The models applied in BI analysis were as follows: K80+I+G4 for LSU and ITS; GTR+G4, F81+I+G4, and JC+I for the first, second, and third codon positions of *tef1*, respectively; and K80+I, HKY+G4, and GTR+G4 for the first, second, and third codon positions of *rpb2*, respectively. The ML analysis was conducted using RAxML-NG v. 1.2.2 (Kozlov et al. 2019), with statistical support at nodes calculated by non-parametric bootstrapping (BS) with 1,000 replicates. The BI analysis was conducted with MrBayes v. 3.2.3 (Ronquist et al. 2012), using two parallel runs with four chains each for 10 million generations (sampled every 1,000 generations). The first 25% of sampled trees were discarded as burn-in. The resulting trees were visualized using FigTree v. 1.4.2 (http://tree.bio.ed.ac.uk/software/figtree/) and graphically edited in CorelDRAW X7 (Ottawa, Canada).

**Table 1.** GenBank accession numbers and details of strains used in the phylogenetic analyses.

Species	Strain	Isolation source	Location	GenBank accession numbers			
				LSU	ITS	*tef1*	*rpb2*
*Capnodiumalfenasii*	CBS 146151^T^ = CPC 22666	*Tabebuia* sp.	Brazil	MN749165	MN749233	MN829346	MN829260
*Capnodiumcoffeae*	CBS 147.52 = AFTOL-ID 939	*Coffearobusta*	Zaire	GU214400	DQ491515	DQ471089	KT216519
*Capnodiumgamsii*	CBS 892.73^T^	Sooty mould, on unknown leaf	Sri Lanka	GU301847	MN749237	GU349045	GU371736
*Chaetocapnodiumindonesiacum*	CBS 202.30^T^	*Cameliasinensis*	Indonesia	GU301849	MH855113	GU349060	MN829273
*Chaetocapnodiuminsulare*	CBS 146159^T^ = CPC 19221	*Phylicaarborea*	South Africa	MN749178	MN749248	MN829359	MN829274
*Chaetocapnodiuminsulare*	CBS 146160 = CPC 19223	*Phylicaarborea*	South Africa	MN749179	MN749249	MN829360	MN829275
*Chaetocapnodiuminsulare*	CBS 146161 = CPC 19224	*Phylicaarborea*	South Africa	MN749180	MN749250	MN829361	MN829276
***Chaetocapnodiummagnum***	**CBS 153154^T^ = P0022**	**Resin of Larixdeciduassp.polonica**	**Poland**	**PV583758**	**PV583761**	**PV591868**	**PV591871**
***Chaetocapnodiummagnum***	**CBS 153155 = P0023**	**Resin of *Abiesalba***	**Poland**	**PV583759**	**PV583762**	**PV591869**	**PV591872**
*Chaetocapnodiummicroglobulosum*	IRAN 2466C	*Actinidiadeliciosa*	Iran	MG920043	MG920019	–	–
*Chaetocapnodiummicroglobulosum*	IRAN 2555C	*Citrussinensis*	Iran	MG920044	MG920020	–	–
*Chaetocapnodiumphilippinense*	MFLUCC 12-0110^T^ = CPC 20474	Palm	Philippines	KP744503	MN749251	MN829362	MN829277
*Chaetocapnodiumplacitae*	CBS 124758^T^ = CPC 13706	*Eucalyptusplacita*	Australia	GQ303299	GQ303268	MN829363	MN829278
***Chaetocapnodiumpolonicum***	**CBS 153156^T^ = P0024**	**Resin of *Abiesalba***	**Poland**	**PV583760**	**PV583763**	**PV591870**	**PV591873**
*Chaetocapnodiumsiamense*	MFLUCC 13-0778^T^	Leaves of unidentified plant	Thailand	KP744479	–	–	–
*Chaetocapnodiumsummerellii*	CBS 146157^T^ = CPC 13654	*Eucalyptusplacita*	Australia	MN749176	MN749246	MN829357	MN829271
*Chaetocapnodiumsummerellii*	CBS 146158 = CPC 17368	–	Laos	MN749177	MN749247	MN829358	MN829272
*Chaetocapnodiumtanzanicum*	CBS 145.79^T^	Lichen	Tanzania	MN749182	MN749253	MN829365	MN829280
*Chaetocapnodiumthailandense*	CBS 139619^T^ = MFLUCC 13-0787	Unknown plant	Thailand	MN749183	MN749254	MN829366	MN829281
*Chaetocapnodiumzapotae*	CM-CNRG 938^T^	Fruit of *Manilkarazapota*	Mexico	MW258621	MW258620	–	–
*Heteroconiumcitharexyli*	BPI 443925^IT^	*Citharexylumilicifolium*	Ecuador	HM628775	HM628776	–	–
*Leptoxyphiumcitri*	CBS 451.66^T^	*Citrussinensis*	Spain	KF902094	MN749266	GU349039	GU371727
*Leptoxyphiummadagascariense*	CBS 124766^T^ = CPC 14623	*Eucalyptuscamaldulensis*	Madagascar	MH874923	MH863407	MN829380	MN829296

Sequences and details of strains obtained in this study are shown in bold. Abbreviations: T: ex-holotype; IT: ex-isotype; –: indicates unavailable data or sequence.

## ﻿Results

**Figure 1.**
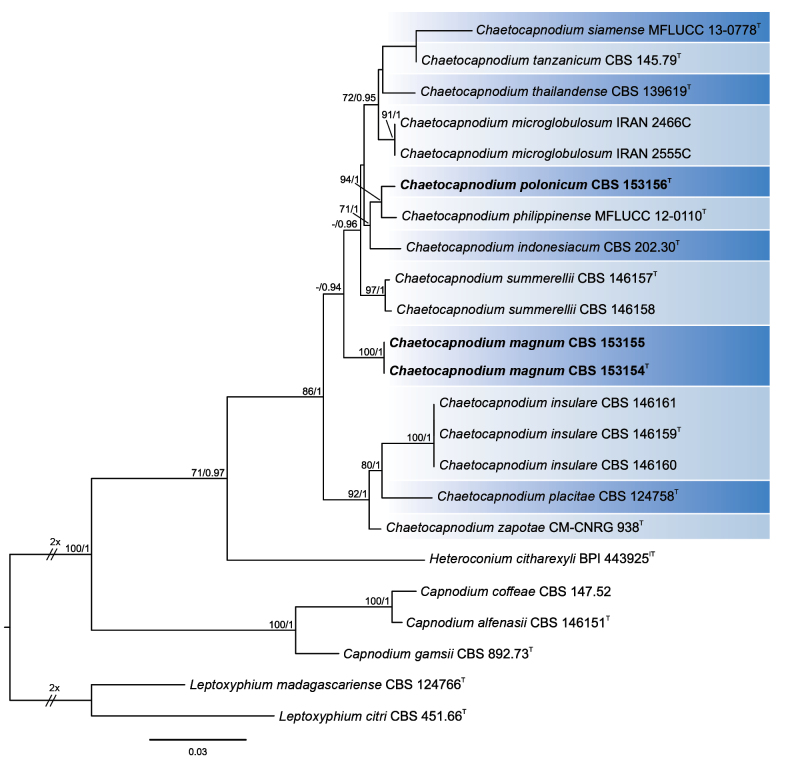
Maximum likelihood consensus tree inferred from the combined LSU, ITS, *tef1*, and *rpb2* multiple sequence alignment of representatives of the genus *Chaetocapnodium* and selected members of closely related genera. The positions of strains of new species *Chaetocapnodiummagnum* and *Ch.polonicum* are indicated in bold. Maximum likelihood bootstrap (MLB) support values ≥ 70% and Bayesian posterior probabilities (BPP) ≥ 0.9 are given next to the branches (MLB/BPP). Ex-type and ex-isotype strains are indicated with T and IT, respectively. The scale bar represents the average number of substitutions per site.

The multi-gene alignment length was 3,267 bp (LSU: 836, ITS: 471, *tef1*: 912, *rpb2*: 1,048), including gaps. The phylogenetic analyses included 21 ingroup taxa, with *Leptoxyphiumcitri* (CBS 451.66) and *Leptoxyphiummadagascariense* (CBS 124766) as outgroup taxa. The ML and BI phylogenetic trees were compared visually for topological conflicts. Both ML and BI analyses returned the same topology, and the ML tree was used to present the results. Maximum likelihood bootstrap (MLB) support values above 70% and Bayesian posterior probabilities (BPP) values above 0.9 are shown for the supported branches (Fig. 1). One of the analyzed strains (CBS 153156), assigned to the new species *Chaetocapnodiumpolonicum*, formed a well-supported (MLB = 94, BPP = 1) sister lineage to *Ch.philippinense*, and together they formed a sister group to *Ch.indonesiacum* (MLB = 71, BPP = 1). Two of the analyzed strains (CBS 153154 and CBS 153155), assigned to the new species *Chaetocapnodiummagnum*, formed a distinct lineage (MLB = 100, BPP = 1) that was placed (MLB = 46, BPP = 0.94) between a lineage containing *Ch.insulare*, *Ch.placitae*, and *Ch.zapotae* and a lineage containing *Ch.indonesiacum*, *Ch.microglobulosum*, *Ch.philippinense*, *Ch.polonicum*, *Ch.siamense*, *Ch.summerellii*, *Ch.tanzanicum*, and *Ch.thailandense* (Fig. 1).

### ﻿Taxonomy

#### ﻿ Chaetocapnodium magnum

Taxon classificationFungiCapnodialesCapnodiaceae

Czachura, Janik & Piątek sp. nov.

566CFA80-301C-5D14-A79F-01FE3A2B7777

859572

Figs 2 , 3

##### Etymology.

The name refers to the pycnidial size, which is the largest among all currently known species of this genus.

##### Typus.

Poland • Podkarpackie Province, Krosno County, the Modrzyna Reserve, on resin of Larixdeciduassp.polonica, 22 Oct. 2020, leg. P. Czachura (holotype: KRAM F-60024; culture ex-type: CBS 153154 = P0022).

##### Description.

Hyphae hyaline (when young), pale brown or brown, smooth or verruculose, branched, septate, mostly constricted at septa, sometimes anastomosing, 2–7.5 μm wide. Pycnidia globose, subglobose, or pyriform, formed intercalary or terminal on hyphae; peridium composed of pale brown, brown, or pale olivaceous pseudoparenchymatous cells arranged in textura angularis, 44.5–209 × 35.5–158 μm. Setae absent or present, aseptate or one-septate, dark brown, rarely branched, 10.5–26 μm long. Ostiole absent or present, not well-developed or well-developed. Conidia release from ostiole or by rupture of peridium, hyaline or subhyaline, aseptate, globose or subglobose, smooth, 2.5–3.7 × 2.4–3.2 μm.

**Figure 2.**
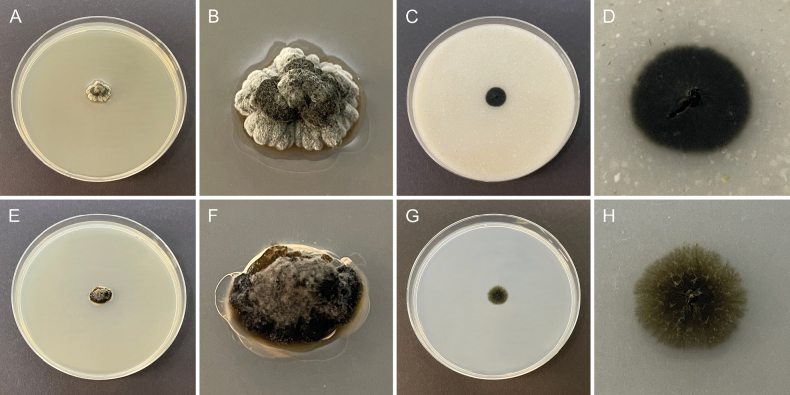
Morphology of cultures of *Chaetocapnodiummagnum* (CBS 153154) after 2 weeks at 25 °C. **A**. **B.** Colony on MEA; **C**, **D.** Colony on OA; **E**, **F.** Colony on PDA; **G**, **H.** Colony on SNA.

##### Culture characteristics.

Colonies on MEA convex, greenish black, greenish grey, or pale greenish grey, reaching 16 mm diam. after 2 weeks at 15 °C and 13 mm diam. after 2 weeks at 25 °C, margin lobate, reverse greenish black. Colonies on OA flat, greenish black, reaching 9 mm diam. after 2 weeks at 15 °C and 12 mm diam. after 2 weeks at 25 °C, margin nearly entire, reverse greenish black. Colonies on PDA convex, olivaceous black with fluffy, grey olivaceous aerial mycelium, reaching 15 mm diam. after 2 weeks at 15 °C and 12 mm diam. after 2 weeks at 25 °C, margin irregular from undulate to lobate, reverse olivaceous black. Colonies on SNA flat, grey olivaceous, with sparse aerial mycelium, reaching 11 mm diam. after 2 weeks at 15 °C and 11 mm diam. after 2 weeks at 25 °C, margin fimbriate, reverse grey olivaceous.

**Figure 3.**
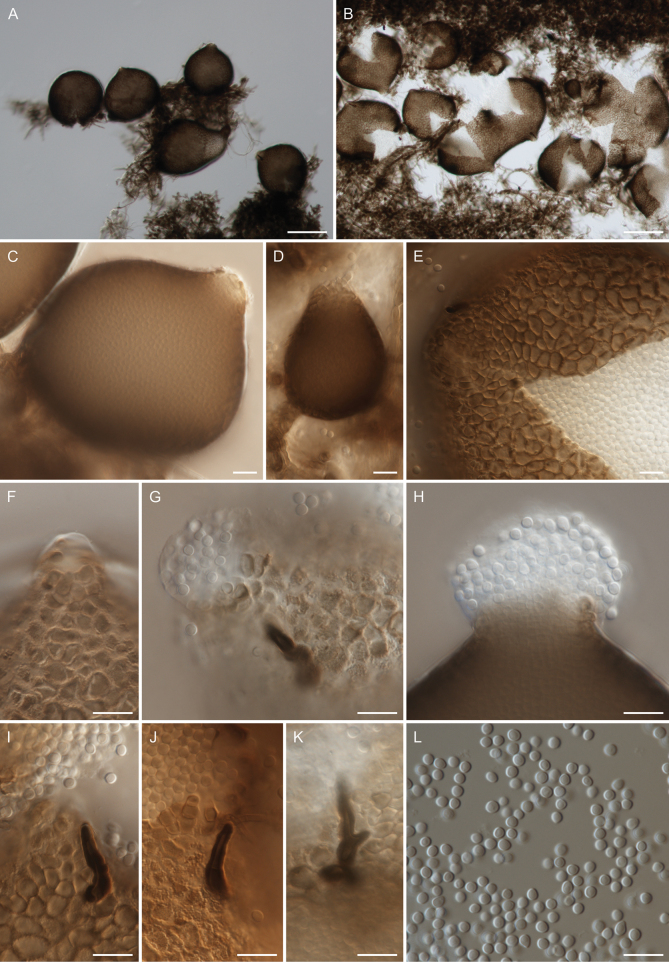
Morphology of *Chaetocapnodiummagnum* (CBS 153154). **A–D.** Pycnidia; **E.** Ruptured peridium with released conidia; **F.** Ostiole not well-developed; **G**, **H.** Ostioles well-developed with released conidia; **I–K.** Setae; **L.** Conidia. Scale bars: 100 µm (**A, B**); 10 µm (**C–L**).

##### Additional specimen examined.

Poland • Świętokrzyskie Province, Kielce County, the Świętokrzyski National Park, the strict protection area Psarski Dół, on resin of *Abiesalba*, 16 Oct. 2020, leg. P. Czachura (KRAM F-60025; culture: CBS 153155 = P0023).

##### Notes.

*Chaetocapnodiummagnum* formed a highly distinct phylogenetic lineage within representatives of the genus *Chaetocapnodium*. Morphologically, *Chaetocapnodiummagnum* differs from the rest of the *Chaetocapnodium* species by having the largest pycnidia amongst all currently known *Chaetocapnodium* species. Moreover, all other species have straight setae in contrast to the occasionally branched setae observed in *Chaetocapnodiummagnum*, a feature that is observed in the genus *Chaetocapnodium* for the first time.

#### ﻿ Chaetocapnodium polonicum

Taxon classificationFungiCapnodialesCapnodiaceae

Czachura, Janik & Piątek sp. nov.

9995BC01-1DEA-5D0D-B026-9F490519878D

859573

Figs 4 , 5

##### Etymology.

The name refers to Poland, where the fungus was collected.

##### Typus.

Poland • Świętokrzyskie Province, Kielce County, the Świętokrzyski National Park, Psarska Góra, on resin of *Abiesalba*, 15 Oct. 2020, leg. P. Czachura (holotype: KRAM F-60026; culture ex-type: CBS 153156 = P0024).

##### Description.

Hyphae hyaline, subhyaline, or pale brown, smooth or slightly verruculose, branched, septate, frequently constricted at septa, anastomosing, 2–6 μm wide. Pycnidia globose, subglobose, or pyriform, formed intercalary or terminal on hyphae; peridium composed of pale olivaceous or pale brown pseudoparenchymatous cells arranged in textura angularis, 32.5–113.5 × 25.5–79.5 μm. Setae absent or present, aseptate or 1–3-septate, brown or deep dark brown, 10–33 μm long. Ostiole absent or present, not well-developed or well-developed. Conidia release from ostiole or by rupture of peridium, hyaline or subhyaline, aseptate, globose or subglobose, smooth, 2.1–3.5 × 1.9–3.1 μm.

**Figure 4.**
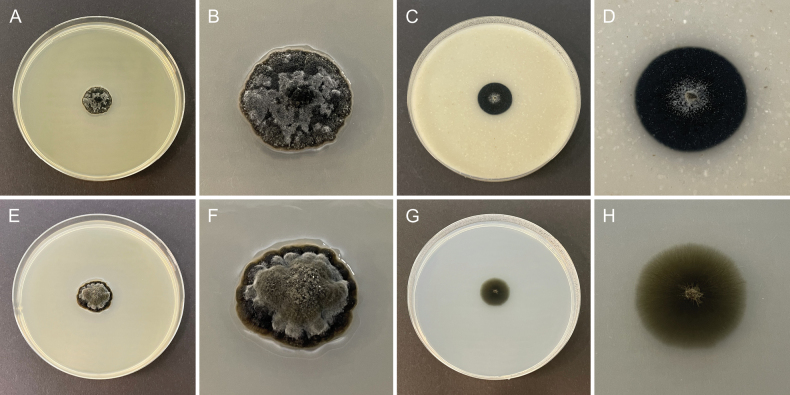
Morphology of cultures of *Chaetocapnodiumpolonicum* (CBS 153156) after 2 weeks at 25 °C. **A**, **B.** Colony on MEA; **C**, **D.** Colony on OA; **E**, **F.** Colony on PDA; **G**, **H.** Colony on SNA.

##### Culture characteristics.

Colonies on MEA slightly convex, dark iron grey or greyish white, reaching 14 mm diam. after 2 weeks at 15 °C and 16 mm diam. after 2 weeks at 25 °C, margin slightly crenate, reverse dark iron grey. Colonies on OA flat, dark iron grey with greyish white center, reaching 10 mm diam. after 2 weeks at 15 °C and 19 mm diam. after 2 weeks at 25 °C, margin entire, reverse dark iron grey. Colonies on PDA convex, dark iron grey, greyish white, or grey olivaceous, reaching 14 mm diam. after 2 weeks at 15 °C and 19 mm diam. after 2 weeks at 25 °C, margin lobate, reverse dark iron grey. Colonies on SNA flat, grey olivaceous, reaching 12 mm diam. after 2 weeks at 15 °C and 17 mm diam. after 2 weeks at 25 °C, margin nearly entire to slightly fimbriate, reverse grey olivaceous.

**Figure 5.**
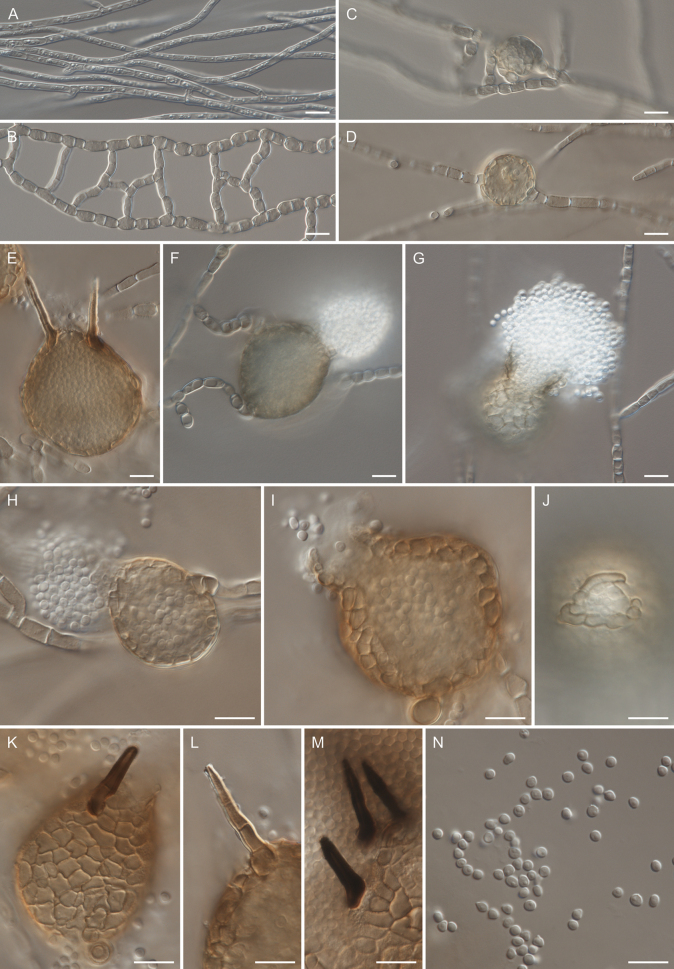
Morphology of *Chaetocapnodiumpolonicum* (CBS 153156). **A.** Hyphae; **B.** Anastomosing hyphae; **C**, **D.** Pycnidia formed intercalary on hyphae; **E.** Pycnidium with setae; **G–I.** Pycnidia with released conidia through ostioles; **J.** Well-developed ostiole; **K–M.** Setae; **N.** Conidia. Scale bars: 10 μm.

##### Notes.

*Chaetocapnodiumpolonicum* is closely related to *Chaetocapnodiumphilippinense* and *Chaetocapnodiumindonesiacum*. It is not possible to compare the morphology of *Ch.polonicum* with *Ch.philippinense* since the former is known only from its asexual morph and the latter from its sexual morph (Liu et al. 2015; Abdollahzadeh et al. 2020). However, *Ch.polonicum* differs from *Ch.philippinense* in LSU (2 bp), ITS (2 bp), *tef1* (8 bp), and *rpb2* (16 bp) sequences, which is an adequate genetic distance for distinguishing species in the genus *Chaetocapnodium* (see Discussion). It is also worth noting that, although not directly comparable between asexual and sexual morphs, setae in the sexual morph of *Ch.philippinense* are up to 90 μm long (measurements taken from illustrations of this species in the original publication: Liu et al. 2015). Thus, they are significantly longer than setae in the asexual morph of *Ch.polonicum*.

Morphologically, *Chaetocapnodiumpolonicum* is significantly different from *Ch.indonesiacum* (Abdollahzadeh et al. 2020). *Chaetocapnodiumpolonicum* produces pycnidia (reaching 113.5 μm) and the longest setae (reaching 33 μm long) observed in asexual morphs (pycnidia) of all described *Chaetocapnodium* species, in contrast to *Ch.indonesiacum*, which has significantly smaller pycnidia ((20–)25–35(–48) μm) and lacks setae. Moreover, *Ch.polonicum* forms well-developed ostioles (if present), in contrast to *Ch.indonesiacum*, in which ostioles are not well-developed (if present), and *Ch.polonicum* produces slightly larger conidia (reaching 3.5 μm) than *Ch.indonesiacum* (reaching 2.8 μm). Finally, both species significantly differ in growth rate; colonies of *Ch.polonicum* reached 16 mm diam. after 2 weeks at 25 °C, in contrast to colonies of *Ch.indonesiacum* that reached 43 mm diam. in the same conditions.

## ﻿Discussion

In this study, two new species of *Chaetocapnodium*—*Ch.magnum* and *Ch.polonicum* were characterized and described. *Chaetocapnodiummagnum* constitutes a phylogenetically and morphologically distinct lineage within the genus *Chaetocapnodium* (Figs 1–3), while *Ch.polonicum* is closely related to *Ch.philippinense* and *Ch.indonesiacum* (Fig. 1). However, it should be noted that the latter three species are separated by small genetic differences. *Chaetocapnodiumpolonicum* differs from *Ch.philippinense* by 2 bp in ITS and 2 bp in LSU, as well as by 1 bp in ITS and 2 bp in LSU from *Ch.indonesiacum*. The phylogenetic distance between *Ch.philippinense* and *Ch.indonesiacum* is 1 bp and 2 bp in ITS and LSU, respectively. Generally, as in many other fungi, ITS and LSU sequences alone are not good markers for separating species in the genus *Chaetocapnodium* (Abdollahzadeh et al. 2020; this study). They should be used in combination with protein-coding genes, which appear to be better markers for phylogenetic studies and species delimitation in this genus. *Chaetocapnodiumpolonicum* differs in *tef1* from *Ch.philippinense* and *Ch.indonesiacum* by 8 bp and 12 bp, respectively. This corresponds with differences between *Ch.philippinense* and *Ch.indonesiacum* or between *Ch.tanzanicum* and *Ch.thailandense*, which are 10 bp and 17 bp, respectively. Differences in the *rpb2* sequences between *Ch.polonicum* and *Ch.philippinense* seem relatively low compared to differences among other species. *Chaetocapnodiumpolonicum* differs from *Ch.philippinense* by 16 bp, whereas differences between other species are larger, for example, 38 bp between *Ch.polonicum* and *Ch.indonesiacum*, 40 bp between *Ch.philippinense* and *Ch.indonesiacum*, and 37 bp between *Ch.tanzanicum* and *Ch.thailandense*. However, it should be noted that *rpb2* sequences are identical in different strains belonging to the same species (e.g., *Ch.insulare*, *Ch.magnum*, *Ch.summerellii*). In such a case, the divergence of *rpb2* sequences between *Ch.polonicum* and *Ch.philippinense* seems adequate to support the recognition of *Ch.polonicum* as a novel species. In summary, *Ch.polonicum* constitutes a distinct species, differing phylogenetically and ecologically, and occurring in a different geographical zone compared to its close relatives *Ch.philippinense* and *Ch.indonesiacum*. Moreover, it is morphologically significantly different from *Ch.indonesiacum*.

The discovery and description of two new species from conifer resins are important in the context of ecology and lifestyle in the genus *Chaetocapnodium*. Previously known *Chaetocapnodium* species were reported as sooty moulds that inhabit sugary exudates called honeydew, excreted by sap-feeding insects (Batista and Ciferri 1963; Cheewangkoon et al. 2009; Liu et al. 2015; Abdollahzadeh et al. 2020; Khodaparast et al. 2020; Navarro De La Fuente et al. 2022). Thus, the newly described *Ch.magnum* and *Ch.polonicum*, found on resin flows, reveal adaptations distinct from other species of the genus. In addition to their different lifestyles, the species examined in this study occur in different geographical locations. Almost all *Chaetocapnodium* species have been reported from tropical or subtropical regions, with the exception of *Ch.insulare*, which was reported from a colder climate—the sub-Antarctic Marion Island in the Southern Hemisphere (Batista and Ciferri 1963; Cheewangkoon et al. 2009; Liu et al. 2015; Abdollahzadeh et al. 2020; Khodaparast et al. 2020; Navarro De La Fuente et al. 2022). Based on current data, *Ch.magnum* and *Ch.polonicum*, together with *Ch.insulare*, are non-tropical species. However, *Ch.insulare* was found exclusively in the Southern Hemisphere, distinguishing it from *Ch.magnum* and *Ch.polonicum*, which are known from the Northern Hemisphere (Abdollahzadeh et al. 2020). Moreover, both species described in this study were found on gymnosperms, in contrast to other *Chaetocapnodium* species, which were found almost exclusively on angiosperms (Batista and Ciferri 1963; Cheewangkoon et al. 2009; Liu et al. 2015; Abdollahzadeh et al. 2020; Khodaparast et al. 2020; Navarro De La Fuente et al. 2022). One species, *Ch.tanzanicum*, occurred on lichen, but based on the general context of the study by Abdollahzadeh et al. (2020), it is suspected that the substrate was covered by honeydew. *Chaetocapnodiummagnum* and *Ch.polonicum* constitute an interesting finding not only at the genus level but also in the context of the ecology of the whole Capnodiales s. str. The order is a rare example of a higher taxonomic lineage highly specialized to a particular habitat and lifestyle, with only a few exceptions (Sutton and Pascoe 1987; Decock 2005; Crous et al. 2015, 2020, 2021; Jitjak and Sanoamuang 2019; Abdollahzadeh et al. 2020). Moreover, most representatives of the order Capnodiales s. str. occur in tropical or subtropical regions on angiosperms, and only a few species have been found in colder regions or on gymnosperms (Hughes 1976; Abdollahzadeh et al. 2020). In summary, the resinicolous *Ch.magnum* and *Ch.polonicum* represent a new ecological niche within the genus *Chaetocapnodium* and the entire order Capnodiales s. str. It should be noted that Hughes (1968, 1976) considered resinicolous *Strigopodiabatistae* and *S.resinae* (= *Capnodiumresinae*) as sooty moulds. *Strigopodia* belongs to Euantennariaceae within Capnodiales s. lat. (Chomnunti et al. 2014; Hyde et al. 2024), but the exact phylogenetic placement of this genus is unknown due to the lack of cultures and DNA sequences.

Resin flows represent a unique habitat in the natural environment, characterized by harsh conditions for microorganisms due to the chemical composition of resins (Langenheim 2003; Cabrita 2018; Czachura and Janik 2025). Despite this, resin flows are inhabited by diverse microorganisms such as bacteria, fungi, and myxomycetes (Vilanova et al. 2014; Mitchell 2021; Gøtzsche et al. 2024), which are probably extremophilic. It is interesting that microorganisms living there are often highly specific to resinous substrates (Rikkinen et al. 2016; Beimforde et al. 2017a; Gøtzsche et al. 2024). Such microorganisms are largely unexplored, including fungi (Mitchell 2021). Most known resinicolous fungi belong to several taxonomic groups such as the classes Eurotiomycetes (Chaetothyriales, Mycocaliciales), Orbiliomycetes (Orbiliales), Leotiomycetes (Helotiales, Leotiales), and Xylonomycetes (syn. Sareomycetes) (Xylonales (syn. Sareales)), as well as some scattered phylogenetic lineages (Rikkinen and Poinar 2000; Tuovila et al. 2013; Beimforde et al. 2017a, 2017b, 2020; Baral et al. 2020; Hashimoto et al. 2021; Mitchell 2021; Mitchell et al. 2021; Czachura and Janik 2025). However, very little data are available for resinicolous fungi in the class Dothideomycetes. To date, only a few members of the Dothideomycetes residing in the order Mytilinidiales have been well documented from resins (Lohman 1933; Speer 1986; Mitchell 2021; Czachura and Janik 2024; Gøtzsche et al. 2024). Several recent studies, however, indicate that fungi from the class Dothideomycetes are more common on conifer resins than previously thought (Crous et al. 2024, 2025). In this study, novel taxa of Dothideomycetes inhabiting resin flows were found and described. The discovery of two new *Chaetocapnodium* species from conifer resins provides new insights into fungal communities living on conifer resin flows, as well as into resinicolous fungi residing in Dothideomycetes.

## Supplementary Material

XML Treatment for Chaetocapnodium magnum

XML Treatment for Chaetocapnodium polonicum

## ﻿Additional information

### Use of AI

No use of AI was reported.

### Author ORCIDs

Paweł Czachura https://orcid.org/0000-0002-3562-8776

Paulina Janik https://orcid.org/0000-0002-4106-636X

Marcin Piątek https://orcid.org/0000-0002-4968-2861

### Data availability

All data supporting the findings of this study are available in the GenBank nucleotide database (https://www.ncbi.nlm.nih.gov/genbank/), the culture collections, and the fungal herbarium, as shown in Table 1 and in the main text.
